# The Psychological Inflexibility in Pain Scale (PIPS) in Brazilian patients with chronic cancer pain: translation, cross-cultural adaptation, and validation study

**DOI:** 10.1590/1516-3180.2025.0018.R1.08092025

**Published:** 2025-12-15

**Authors:** Sergio Paulo Mariano de Aguiar, Daniela Bassi-Dibai, Letícia Padilha Mendes, Naara Rayane Moura Cutrim, César Leonardo Ribeiro Guedes, Jocassia Silva Pinheiro, Daniel Nunes Morais, Carlos Wagner de Sousa Campos, Plínio da Cunha Leal, Almir Vieira Dibai

**Affiliations:** I Master’s student, Programa de Pós-Graduação em Saúde do Adulto, Universidade Federal do Maranhão (UFMA), São Luís (MA), Brazil.; II Professor, Programa de Pós-Graduação em Biociências Aplicadas à Saúde, Universidade Ceuma, São Luís (MA), Brazil.; III Master’s student, Programa de Pósgraduação em Educação Física, Universidade Federal do Maranhão (UFMA), São Luís (MA), Brazil.; IV Undergraduate student, Departamento de Enfermagem, Universidade Federal do Maranhão (UFMA), São Luís (MA), Brazil.; V Master’s student, Programa de Pós-Graduação em Educação Física, Universidade Federal do Maranhão (UFMA), São Luís (MA), Brazil.; VI Doctoral student, Programa de Pós-Graduação em Reabilitação e Desempenho Funcional, Faculdade de Medicina de Ribeirão Preto, Universidade de São Paulo (USP), Ribeirão Preto (SP), Brazil.; VII Master’s student, Programa de Pós-Graduação em Saúde do Adulto, Universidade Federal do Maranhão (UFMA), São Luís (MA), Brazil.; VIII Undergraduate student, Departamento de Educação Física, Universidade Federal do Maranhão (UFMA), São Luís (MA), Brazil.; IX Professor, Programa de Pós-Graduação em Saúde do Adulto; Programa de Pós-Graduação em Educação Física, Universidade Federal do Maranhão (UFMA), São Luís (MA), Brazil.; X Departamento de Educação Física; Programa de Pós-Graduação em Saúde do Adulto; Programa de Pós-Graduação em Educação Física, Universidade Federal do Maranhão (UFMA), São Luís (MA), Brazil.

**Keywords:** Cancer pain, Surveys and questionnaires, Chronic pain, Cognitive fusion, Avoidance, Psychological inflexibility

## Abstract

**BACKGROUND:**

The Psychological Inflexibility in Pain Scale (PIPS) was developed to measure avoidance and cognitive fusion.

**OBJECTIVES:**

To translate, cross-culturally adapt, and analyze the measurement properties of the Psychological Inflexibility in Pain Scale (PIPS) in Brazilian patients with chronic cancer pain.

**METHODS:**

Questionnaire translation, cross-cultural adaptation, and validation studies were conducted in two hospitals in northeastern Brazil. The measurement properties tested included structural validity, construct validity, reliability, and internal consistency. The following assessment instruments were used in addition to the PIPS: Pain Catastrophizing Scale (PCS), Barthel Index, Edmonton Symptom Assessment Scale (ESAS), and Hospital Anxiety and Depression Scale (HADS).

**RESULTS:**

The study sample consisted of 122 patients, most of whom were women (65.6%) with a mean age of 49 years. Most patients had uterine cancer (23%) and leukemia (9.8%). We identified problems in the two-dimensional structure of the PIPS by presenting three inadequate fit indices. Adequate reliability was observed in both domains. Regarding the avoidance domain, there was a correlation with a magnitude > 0.30 with the depression domain of the HADS, and correlations with a magnitude < 0.30 with the anxiety domain of the HADS, the PCS domains, and the Barthel Index. The cognitive fusion domain did not correlate with any of these scales (P > 0.05). No ceiling or floor effects were observed.

**CONCLUSION:**

The Brazilian version of the PIPS is reliable; however, the instrument does not have a valid internal structure and the cognitive fusion domain is not a valid construct.

## INTRODUCTION

 Cancer pain affects approximately half of patients with cancer at different stages of the disease: 50.7% at any stage, 55% during treatment, and 66.4% in terminal, advanced, or metastasized stages. Even after the cancer has been cured, pain is still present in 39.3% of patients.^1^ To assess this pain, one-dimensional scales such as the Visual Analog Scale, Numerical Pain Scale, and Wong-Baker Faces Pain Rating Scale are commonly used.^2^ However, it is crucial also to consider the factors that can intensify or alleviate pain, such as physical, psychological and social aspects, for a broader understanding of the cancer pain phenomenon.^3^

 The Psychological Inflexibility in Pain Scale (PIPS) is a valuable tool based on acceptance and commitment to therapy. Psychologically flexible individuals can pursue their goals and values despite experiencing pain. The PIPS was developed to measure inflexibility in patients with chronic pain, regardless of comorbidities, by assessing two distinct domains: avoidance and cognitive fusion.^4^

 This scale has already been validated in several languages and cultures, including Spanish, German, Persian, Japanese and Chinese.^5 ,9^ The Chinese version of the PIPS was adapted for 389 patients with cancer, demonstrating adequate psychometric properties.^8^ Thus, its use for pain management in patients with cancer can contribute significantly to the treatment of these individuals. 

 Therefore, considering the importance of psychological inflexibility in chronic pain, conducting an adaptation study of the PIPS into Brazilian Portuguese is justifiable since this still needs to be done. This study aimed to translate, culturally adapt, and evaluate the measurement properties of the PIPS for Brazilian patients with chronic cancer pain. 

## METHODS

### Study design

 A study was carried out to translate, cross-culturally adapt, and validate an evaluation scale based on the guidelines of the Consensus-based Standards for selecting health Measurement Instruments (COSMIN)^10^ and the Guidelines for cross-cultural adaptation of self-report measures.^11^ Permission to use the instrument was granted via e-mail (Dr. Rikard K. Wicksell). 

 The study was conducted at the Maranhão Oncology Hospital and Aldenora Bello Hospital in São Luís, Northeast Brazil. The University Research Ethics Committee approved this study (approval no. 5.232.253). 

### Sample

 The sample size was calculated based on the COSMIN guidelines: seven times the number of items in the questionnaire, if this value is not less than 100. Given that the PIPS contains 12 items, the minimum sample size was 100 patients.^10^

 The inclusion criteria for the study were as follows: cancer pain for at least 3 months; age 18 years or older, both sexes, ability to read and understand Brazilian Portuguese, diagnosis of cancer confirmed by biopsy, and awareness of cancer diagnosis. 

 The following patients were excluded from the study: those diagnosed with severe cognitive or psychiatric disorders, those who could not complete the questionnaires, and those without pain when filling in the questionnaires. 

### Translation and cross-cultural adaptation

 The process of translation and cross-cultural adaptation of the PIPS followed the criteria of Beaton et al.^11^ as described below.

1. Translation: Two independent translators with Portuguese as their mother tongue and fluency in English translated the PIPS into Brazilian Portuguese. One translator was from the health field. Each translator then produced a translation report.
2. Synthesis of translations: The two translators held discussions and revisions until they reached a single version of the PIPS by consensus under the observation of one of the researchers.
3. Back translation: Two translators translated the questionnaire back into their original language without prior knowledge of the original version. These translators have English as their mother tongue but are fluent in Brazilian Portuguese and are not from the health field.
4. Analysis by a committee of experts: This committee included four translators, three physiotherapists, and one physician. This group reviewed all translated and back-translated versions to correct possible discrepancies and arrived at the final version of the questionnaire.
5. Testing the pre-final version: The pre-final version was administered to 30 patients with cancer. This phase aimed to establish the degree of understanding of the items in the pre-final version of the PIPS. If the comprehension of each item was greater than 80%, the pre-final version was defined as the final version. If an item had less than 80% comprehension, these items were modified and tested on a new sample of 30 participants.
6. Final version: After all the stages, the research coordinator approved the final version of the PIPS in Brazilian Portuguese.

### INSTRUMENTS

 Initially, sociodemographic data and clinical characteristics of the patients were collected, including primary diagnosis, presence of comorbidities, date of diagnosis, presence of metastasis, length of treatment, and signs and symptoms. Subsequently, the evaluation scales were applied. 

### Psychological Inflexibility in Pain Scale (PIPS)

 The PIPS was developed by Wicksell et al.^12^ and has 12 items containing seven response options: 1. never true; 2. very rarely true; 3. seldom true; 4. sometimes true; 5. often true; 6. almost always true; and 7. always true. The PIPS has two domains: avoidance (items 1, 2, 4, 5, 7, 8, 10, and 11), and cognitive fusion (items 3, 6, 9, and 12). The avoidance domain relates to the patient’s tendency to engage in certain behaviors to avoid pain and suffering, whereas the cognitive fusion domain assesses the frequency with which each individual manifests an action in the face of these thoughts as if they were true. The scores for the avoidance and cognitive fusion domains ranged from 8 to 56 and 4 to 28, respectively. 

### Pain Catastrophizing Scale (PCS)

 The PCS consists of 13 items that assess pain catastrophizing behavior. It consists of three subscales: hopelessness, magnification, and rumination. Patients were required to answer items according to their thoughts and feelings when experiencing pain. The items were classified on a 5-point scale, divided into not at all (score 0) to all the time (score 4).^13^ Thus, the total score for domain rumination ranged from 0 to 16, magnification ranged from 0 to 12, and helplessness ranged from 0 to 24 points. Higher scores indicated greater catastrophizing. The PCS was validated in Brazil for patients with chronic pain by Sehn et al.^14^

### Hospital Anxiety and Depression Scale (HADS)

 The HADS consists of 14 items, seven of which are used to assess anxiety and seven to measure depression and was validated for Brazil by Botega et al.^15^ Each item is scored on a scale from 0 to 3, with a total of 21 points for each scale. The higher the score, the greater the signs of anxiety or depression. 

### Barthel Index

 The Barthel Index was validated for Brazil by Barros et al.,^16^ composed of 10 items that assess patients’ level of functional independence in daily activities, with scores ranging from 0 to 100. Higher scores indicated greater functional independence. 

### Edmonton Symptom Assessment System (ESAS)

 The ESAS assesses pain, activity, nausea, depression, anxiety, drowsiness, appetite, well-being, and shortness of breath. For each symptom, it is possible to sign a scale ranging from 0 to 10, where zero represents the absence of the symptom and 10 describes the symptom in its strongest manifestation. This scale was validated in Brazil by Monteiro et al.^17^ In this study, the ESAS was used only to characterize the sample. 

### Statistical analysis

 Sociodemographic data were described as means and standard deviations (quantitative data) or absolute numbers and percentages (qualitative data). The internal consistency of each domain was calculated using Cronbach’s alpha, considering a range between 0.7 and 0.95 to be adequate values.^18^

 Reliability was assessed using a test-retest model. The intraclass correlation coefficient (ICC), 95% confidence interval (95% CI), standard error of measurement (SEM), and minimum detectable difference (MDD) were used to assess the reliability of the scores for each domain of the PIPS. ICC values greater than or equal to 0.75 was acceptable.^19^

 Structural validity was analyzed using confirmatory factor analysis considering the original proposal of two domains and 12 items.^12^ We used the software R Studio (Boston), with lavaan and SemPlot packages, and with the implementation of a polychoric matrix and the robust diagonally weighted least squares extraction method.^20,21^ The following cutoff values were considered adequate for the fit indices: chi-square/degree of freedom (DF) < 3; comparative fit index (CFI) and Tucker-Lewis index (TLI) > 0.9; and root mean square error of approximation (RMSEA) and standardized root mean squared residual (SRMR) < 0.08.^22 ,23^ Factor loadings were considered adequate when ≥ 0.4.^24^ We used modification indices (MI) to analyze the model, considering a value > 10 as the cutoff point for identifying a problem in the model.^25^

 For construct validity using correlations between instruments, the normality of the data was initially checked using the Kolmogorov–Smirnov test. Subsequently, Spearman’s correlation coefficient (ρ) was used to determine the magnitude of the correlation between the PIPS and other measurement instruments. Interpretation of the magnitude of the correlations followed the following criteria: correlations with instruments measuring similar constructs should be ≥ 0.5; correlations with instruments measuring related but different constructs should be between 0.3 and 0.5; and correlations with instruments measuring unrelated constructs should be < 0.3.^10^ This study hypothesizes that the domains of the PIPS show a significant correlation magnitude of < 0.3. 

 The effects of the floor and ceiling were also evaluated in this study. These effects occurred when more than 15% of the study participants (more than 15%) reached the minimum or maximum total score on the questionnaire, indicating a problem in assessing the instrument’s responsiveness. 

 Internal consistency, reliability, and correlations were analyzed using SPSS statistical software (version 17.0, Chicago), and a 5% significance level was adopted. 

## RESULTS

### Cross-cultural adaptation

 It was unnecessary to adapt any terms or expressions to Portuguese during the translation. The pre-final version of the PIPS was administered to 30 patients with cancer-related pain. Only one patient (3.33%) needed help in understanding items 2 and 10 of the PIPS. Therefore, there was acceptable comprehension of the PIPS items (> 80%). 

### Structural validity

**Figure 1:**
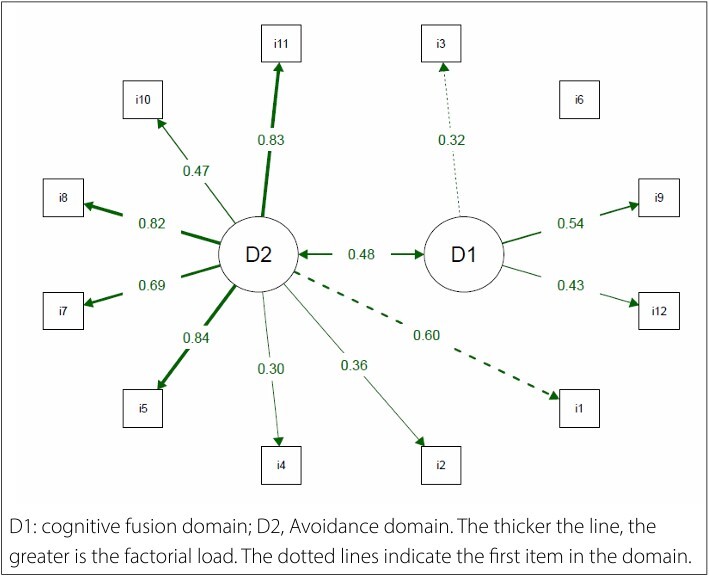
Path diagram of the two-dimensional structure of the Psychological Inflexibility in Pain Scale (PIPS).

 We identified problems with the two-dimensional structure proposed for creating the PIPS. Three fit indices were inadequate (TLI = 0.88, RMSEA = 0.102, SRMR = 0.101), whereas two were adequate (chi-square/DF = 2.2 and CFI = 0.903). The twodimensional structure also showed inadequate factor loadings (less than 0.4) for items 3 (factor loading = 0.32) and 6 (factor loading = 0.09) of the cognitive fusion domain, and for items 2 (factor loading = 0.36) and 4 (factor loading = 0.30) of the avoidance domain, as shown in **Figure 1**. 

 Using MI, we investigated the model’s problems, identifying that item 6 was consistently related to the avoidance domain (MI = 14.408), items 6 and 12 presented redundancy in the patients’ response pattern (MI = 13.502), and items 3 and 6 presented redundancy in the patients’ response pattern (MI = 10.99). 

### Characterization of the sample

 The sample consisted of 122 patients, most of whom were women (65.6%) with an average age of approximately 49 years, were married (50.8%), and had completed elementary school (46.7%). Regarding the type of cancer, most were uterine cancer (23%) or leukemia (9.8%). Among the participants’ characteristics, there was a predominance of cases without metastasis (80.3%) and receiving curative care (95.9%). Other information are described in **Table 1** and **Table 2**. 

**Table 1:** Personal and social characteristics of the patients included in the study (n = 122)

**Variables**		**Number (%)**
Sex		
	*Male*	42 (34.4%)
	*Female*	80 (65.6%)
Marital status		
	*Married*	62 (50.8%)
	*Divorced*	7 (5.7%)
	*Single*	50 (41%)
	*Widowed*	3 (2.5%)
Scholarity		
	*Primary education*	12 (9.8%)
	*Basic education*	57 (46.7%)
	*High school*	40 (32.8%)
	*Higher education*	13 (10.7%)
Type of cancer		
	*Uterus*	28 (23%)
	*Leukemia*	12 (9.8%)
	*Lymphoma*	9 (7.4%)
	*Breast*	8 (6.6%)
	*Pancreas*	8 (6.6%)
	*Stomach*	8 (6.6%)
	*Lung*	5 (4.1%)
	*Ovary*	5 (4.1%)
	*Multiple myeloma*	5 (4.1%)
	*Penis*	4 (3.3%)
	*Liver*	3 (1.9%)
	*Bones*	2 (1.6%)
	*Vulva*	2 (1.6%)
	*Others*	23 (18.9%)
Metastasis		
	*Yes*	24 (19.7%)
	*No*	98 (80.3%)
Type of treatment		
	*Curative*	117 (95.9%)
	*Palliative*	5 (4.1%)
Treatment modality		
	*Chemotherapy*	49 (40.2%)
	*Surgery*	26 (21.3%)
	*Surgery and medication*	12 (9.8%)
	*Surgery and chemotherapy*	10 (8.2%)
	*Medication*	10 (8.2%)
	*Chemotherapy and radiotherapy*	5 (4.1%)
	*Radiotherapy*	5 (4.1%)
	*Surgery, chemotherapy and radiotherapy*	2 (1.6%)
	*Surgery, chemotherapy and medication*	2 (1.6%)
	*Surgery and radiotherapy*	1 (0.8%)

**Table 2:** Descriptive analysis and scores of the questionnaires and scales used in the study

**Variables**		**Mean (standard deviation)**
Age (years)		49.52 (15.43)
ESAS (score, 0–10)		
	*Pain*	6.68 (2.98)
	*Fatigue*	2.87 (3.3)
	*Nausea*	2.48 (3.19)
	*Sadness*	4.31 (3.67)
	*Anxiety*	4.69 (3.82)
	*Drowsiness*	4.12 (3.79)
	*Lack of appetite*	3.27 (3.4)
	*Shortness of breath*	1.35 (2.48)
	*Lack of well-being*	3.14 (3.25)
Barthel index (score, 0–100)		77.13 (29.02)
HADS (score, 0–21)		
	*Anxiety*	7.26 (4.49)
	*Depression*	6.68 (4.12)
PCS		
	*Helplessness (score, 0–24)*	8.48 (5.79)
	*Magnification (score, 0–12)*	6.56 (4.7)
	*Rumination (score, 0–16)*	9.69 (4.04)
PIPS		
	*Cognitive fusion (score, 4–28)*	23.19 (3.71)
	*Avoidance (score, 8–56)*	34.76 (9.99)

ESAS, Edmonton Symptom Assessment Scale; HADS, Hospital Anxiety and Depression Scale; PCS, Pain Catastrophizing Scale; PIPS, Psychological Inflexibility in Pain Scale.

### Reliability and internal consistency

 As shown in **Table 3**, we observed adequate test-retest reliability and internal consistency values, with an ICC of 0.8 for the cognitive fusion domain and 0.95 for the avoidance domain. Internal consistency was also acceptable, with Cronbach’s alpha of 0.7 and 0.74 for the cognitive fusion and avoidance domains, respectively. 

**Table 3:** Test-retest reliability and internal consistency of the Psychological Inflexibility in Pain Scale (PIPS)

**Measures**	**Domains**	
	**Cognitive fusion**	**Avoidance**
Test, mean (standard deviation)	23.05 (3.26)	37.12 (9.34)
Retest, mean (standard deviation)	23.48 (3.56)	38.28 (8.83)
ICC	0.8	0.95
95% CI of ICC	0.68 to 0.88	0.92 to 0.97
SEM, score (%)	1.54 (6.62%)	2.03 (5.39%)
MDD, score (%)	4.27 (18.35%)	5.63 (14.94%)
Cronbach’s alpha	0.7	0.74

ICC, Intraclass correlation coefficient; CI, Confidence interval; SEM, Standard error of measurement; MDD, Minimum detectable difference.

### Construct validity via hypothesis testing

 Regarding the avoidance domain of the PIPS, there was a correlation with a magnitude > 0.3 with the depression domain of the HADS and correlations with a magnitude < 0.3 with the anxiety domain of the HADS, the domains of the PCS, and the Barthel index (**Table 4**). Unexpectedly, the cognitive fusion domain of the PIPS did not correlate with the instruments used in this study (P > 0.05). 

**Table 4:** Correlation between the domains of the Psychological Inflexibility in Pain Scale (PIPS) and other scales

**Variables**		**Cognitive fusion**		**Avoidance**	
		**ρ**	**P**	**ρ**	**P**
Barthel index		0.056	0.551	−0.196	0.035^*^
HADS					
	*Anxiety*	0.116	0.205	0.237	0.009^*^
	*Depression*	0.018	0.846	0.358	0.001^*^
PCS					
	*Helplessness*	0.084	0.355	0.205	0.023^*^
	*Magnification*	0.081	0.377	0.275	0.002^*^
	*Rumination*	0.113	0.216	0.297	0.001^*^

HADS, Hospital Anxiety and Depression Scale; PCS, Pain Catastrophizing Scale.

* Significant correlation (P < 0.05, Spearman correlation coefficient).

### Floor and ceiling effects

 We did not observe ceiling or floor effects, as in the no-PIPS domain, there were more than 15% maximum or minimum responses. For the cognitive fusion domain, none of the participants reached the minimum score (4 points) and 17 patients (13.9%) reached the maximum score (28 points); for the avoidance domain, one participant (0.8%) reached the minimum score (8 points), and no participant reached the maximum score (56 points). 

## DISCUSSION

 Satisfactory test-retest reliability and internal consistency values were observed, ensuring score stability in the PIPS assessments conducted on different days. However, the two-dimensional internal structure of the PIPS proved inadequate, indicating that the scale items did not accurately reflect the intended domains. Finally, the avoidance domain was a valid construct, whereas the cognitive fusion domain showed no correlation with any of the instruments evaluated in the study. 

 Regarding reliability, we found acceptable ICC values, i.e., 0.8 and 0.95 for the cognitive fusion and avoidance domains, respectively. A Spanish study identified relatively higher reliability values, with an ICC of 0.97 for both domains, in which patients with fibromyalgia were investigated.^7^ Similarly, a Chinese study also found higher values, with an ICC of 0.98 for the avoidance domain and 0.97 for cognitive fusion. The Chinese version assessed patients with chronic cancerrelated pain.^8^ Notably, all the versions mentioned, including the present study, had ICC values within the acceptable range, that is, greater than 0.75. 

 Regarding internal consistency, Cronbach’s alpha values for the avoidance and cognitive fusion domains were 0.74 and 0.7, respectively. The Chinese study obtained a satisfactory Cronbach’s alpha (0.74 for cognitive fusion and 0.88 for avoidance).^8^ Similar to previous studies, the present study showed adequate internal consistency values for the avoidance domain, with Cronbach’s alpha coefficient ranging from 0.89 to 0.95.^5-7 ,9,12,26^ However, in the cognitive fusion domain, internal consistency proved to be inadequate in most versions, with Cronbach’s alpha coefficient below 0.7 in the original, German, Spanish, and Japanese versions.^5-7,27^

 We partially confirmed the hypotheses of this study in terms of construct validity. The avoidance domain showed the expected correlations with anxiety, depression, catastrophizing, and functional independence. However, we identified a problem with the other PIPS domains, as we did not recognize the cognitive fusion construct as valid, given that there was no correlation with the tools used. 

 We observed that most validation studies on the PIPS correlated with the total score of the instrument and did not separate it by the domains of avoidance and cognitive fusion. The Chinese version of the PIPS with patients with cancer found correlation between the total PIPS score and the Acceptance and Action Questionnaire (ρ = 0.54) and the Chronic Pain Acceptance Questionnaire (ρ = −0.41).^8^

 Part of the construct validity was related to the internal structure of the PIPS. In this sense, some studies conducted factor analysis and did not find adequate fit indices to support the structure with two domains and 12 items. The original study^27^ showed residuals in the model that were higher than satisfactory. The Japanese study found inadequate fit indices, and it was only possible to fit the model after adding eight correlations between PIPS items.^6^ The Greek study also found inadequate fit indices for the twodimensional structure of the instrument,^28^ similar to the results of the present study. 

 Contrastingly, the Spanish version found an adequate internal structure but used principal component analysis as a method, which is unsuitable for instruments with a reflective model.^7^ The Chinese study also found an adequate two-dimensional structure but used an extraction method that was not very suitable for factor analysis of instruments with ordinal categorical responses.^8^

 From clinical and research perspectives, the PIPS should be used with caution. Based on the results obtained and those of previous studies, the cognitive fusion construct appears problematic and requires confirmation or correction in studies with robust statistical methodologies. 

 The limitations of this study include the heterogeneity of the cancer types in the sample and the inclusion of a significant proportion of patients without metastasis or in palliative care conditions, typically associated with more severe pain. Additionally, our sample was recruited from a hospital-based oncology service; thus, the evaluative capacity of the PIPS may differ in outpatient or home-based care settings. 

## CONCLUSIONS

 The Brazilian version of the PIPS is reliable. However, the twodimensional structure (cognitive fusion and avoidance domains) does not have a valid internal structure, and the cognitive fusion domain is not a valid construct when analyzed via hypothesis testing. 

## Data Availability

The data that support the findings of this study are available from the corresponding author, Letícia Padilha Mendes, upon reasonable request.
